# Fatal Acute Hypoxemic Respiratory Failure Caused by *Burkholderia thailandensis*, China

**DOI:** 10.3201/eid3107.241920

**Published:** 2025-07

**Authors:** Pei Zhang, Dai Kuang, Shaowen Chen, Wei Liu, Xuehan Duan, Yang Chen, Xuming Wang, Qianfeng Xia, Hua Wu

**Affiliations:** Hainan General Hospital, Affiliated Hainan Hospital of Hainan Medical University, Haikou, China (P. Zhang, Y. Chen, X. Wang, H. Wu); NHC Key Laboratory of Tropical Disease Control, School of Tropical Medicine, Hainan Medical University, Haikou (D. Kuang, W. Liu, Q. Xia); Second Affiliated Hospital of Hainan Medical University, Haikou (S. Chen); Linyi Traditional Chinese Medical Hospital, Linyi, China (X. Duan)

**Keywords:** acute hypoxemic respiratory failure, *Burkholderia thailandensis*, septic shock, melioidosis, *Burkholderia pseudomallei*, bacteria, respiratory infections, China

## Abstract

We report on a patient in China with no underlying illnesses who died of *Burkholderia thailandensis* infection despite timely treatment. This case challenges the perception that *B. thailandensis* is nonlethal or has low virulence. Increased clinical awareness and prompt diagnosis are essential for managing *B. thailandensis* infections and preventing fatal outcomes.

*Burkholderia thailandensis* is commonly regarded as nonpathogenic, whereas *B. pseudomallei* is recognized as the most clinically relevant species known to cause melioidosis (*1*). We describe a rare case of fatal acute hypoxemic respiratory failure and septic shock caused by *B. thailandensis* in a previously healthy person from Hainan Province, China. The Institutional Review Board at Hainan General Hospital approved the study protocol.

A 58-year-old male rice farmer from the central region of Hainan Province was admitted to an emergency department on May 23, 2019. He exhibited an unexplained onset of cough, sputum production, chest tightness, and shortness of breath that had persisted for 8 days. He did not smoke, have a previous family history of those respiratory conditions, or have immune deficiencies. His vital signs and laboratory test results were recorded at admission (Appendix Table 1). His acute physiology and chronic health evaluation II score was 24 and sequential organ failure assessment score was 13 after 1 day of hospitalization. A chest computed tomography scan (Figure) revealed a large high-density shadow in the left lung field and a small effusion in the left pleural cavity, indicating a substantial infection in the left lung. Tigecycline was administered for infection control.

**Figure:**
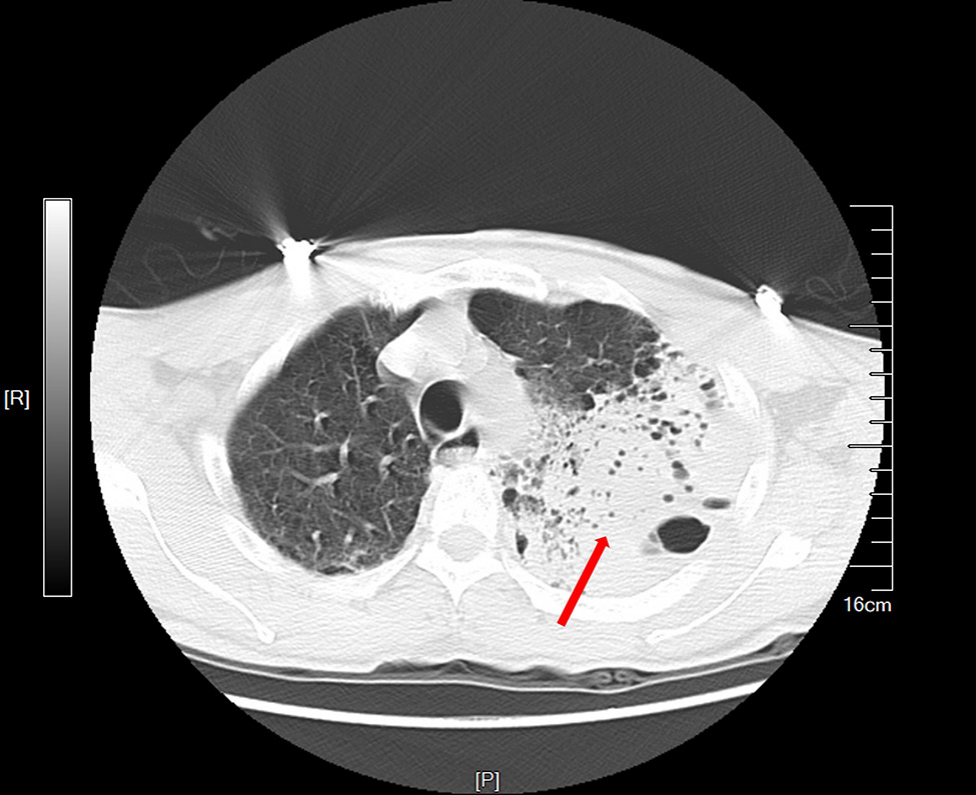
Chest computed tomography scan of patient who died of acute hypoxemic respiratory failure caused by *Burkholderia thailandensis*, China. Scan shows left lung consolidation and pleural effusion. Red arrow indicates a large high-density shadow in the left lung field. Scale indicates actual size of anatomical structures and lesions. P, posterior; R, right.

Bronchoalveolar lavage fluid was submitted for bacterial culture on May 24, 2019. After 48 hours of incubation, colonies consisted of short gram-negative bacilli (Appendix Figure 1). We performed matrix-assisted laser desorption/ionization time-of-flight mass spectrometry profiling (Appendix Figure 2), 16S rRNA-based phylogenetic tree analysis (Appendix Figure 3, panel A), and biochemical identification (Appendix Figure 4). We identified the isolate as *B. thailandensis* strain HNBT001.

Whole-genome sequencing using Illumina (https://www.illumina.com) and Pacific Biosciences (https://www.pacb.com) pipelines revealed the *B. thailandensis* strain consisted of 2 circular sequences: a 3,929,948-bp chromosome and a 2,858,975-bp chromosome (GenBank accession no. GCA_048688115.1), having an average G/C nucleotide content of 67.53%. We used values of digital DNA–DNA hybridization (dDDH) and average nucleotide identity (ANI) to compare the isolated strain with representative genomes of *Burkholderia* spp. in the National Center for Biotechnology Information RefSeq database (https://www.ncbi.nlm.nih.gov/refseq; accessed on June 6, 2024). Using the Type Genome Server (*2*), we found pairwise comparison with a *B*. *thailandensis* reference genome (assembly no. GCF_001718635.1) indicated the genomes of the strain in our study had a dDDH formula d4 value of 89% and an ANI value of 98.9%; both values exceeded the thresholds for prokaryotic species delineation (70% for dDDH) (*3*) and bacterial species differentiation (96% for ANI) (Appendix Figure 5) (*4*). The HNBT001 strain was classified as sequence type 80, previously identified in both non–human-associated (E264) and human-associated (FDAARGOS 240 and 2022Dzh) strains (Appendix Figure 3, panel B).

On the basis of antimicrobial susceptibility test results (Appendix Table 2), we replaced tigecycline with imipenem for infection treatment. The patient’s symptoms lessened after 2 days of imipenem therapy. However, despite a transient improvement in symptoms, his blood oxygenation gradually deteriorated while receiving invasive mechanical ventilation support, leading to multiorgan dysfunction, manifesting as scattered low-density lesions of the liver, splenomegaly, persistent thrombocytopenia, and acute renal failure characterized by substantially elevated serum creatinine levels. In addition, on day 6 of hospitalization, peripheral edema developed. After progressive clinical deterioration, the patient elected to withdraw from further treatment and was discharged after an 8-day hospital admission. During hospitalization, he was treated with imipenem for 5 days. The patient died 3 days after discharge.

*B. thailandensis* is generally considered nonpathogenic and is commonly found in tropical and subtropical environments, particularly in humid soil and water (*5*). In this case, the patient was a farmer who had early clinical manifestations of cough, phlegm, chest tightness, and shortness of breath and was likely exposed to *B. thailandensis* through contact with contaminated soil or water. However, unlike most reported cases, this patient had no previous underlying health conditions.

This case occurred in Hainan Province, located >1,400 km from regions where 2 other cases have been documented in China (*6*,*7*). However, medical experts with extensive experience believed insufficient evidence existed to identify *B. thailandensis* in the first case (*6*,*8*). Through 16S rRNA gene analysis (Appendix Figure 3) and taxonomic comparisons in GenBank (accession no. GCA_011578485.1), we classified the strain in this case as *B. thailandensis*. ​In the previous case involving wound infection (*7*), colonization by nonvirulent *B. thailandensis* could not be definitively excluded. In our case, despite the identification of multiple potential virulence genes in the HNBT001 strain (Appendix Table 3), exact pathogenic mechanisms remain to be elucidated through further research. Because of the high potential for *B. thailandensis* to be misidentified as *B. pseudomallei* (*9*), the prevalence and public health impact of *B. thailandensis* might be considerably underestimated.

In conclusion, contrary to the previously held opinion that *B. thailandensis* is nonpathogenic or of low virulence, we show it can cause severe infections even in immunocompetent patients and potentially fatal outcomes in otherwise healthy persons. We strongly advise medical personnel to place greater emphasis on strengthening biosafety precautions during both laboratory work and clinical treatment involving this underestimated pathogen.

Appendix Additional information for fatal acute hypoxemic respiratory failure caused by *Burkholderia thailandensis*, China.
