# Supplementary material for: Fatal Acute Hypoxemic Respiratory Failure Caused by Burkholderia thailandensis, China
Source: Emerg Infect Dis. 2025 Jul;31(7):1474–6. doi: 10.3201/eid3107.241920 (PMC12205439; doi:10.3201/eid3107.241920)
Supplement: Appendix — Additional information for fatal acute hypoxemic respiratory failure caused by Burkholderia thailandensis, China. [file 24-1920-Techapp-s1.pdf]

Article DOI: https://doi.org/10.3201/eid3107.241920
EID cannot ensure accessibility for supplementary materials supplied by authors. Readers who have difficulty accessing supplementary content should contact the authors for assistance.

Fatal Acute Hypoxemic Respiratory Failure Caused by *Burkholderia thailandensis*, China

Appendix

Appendix Table 1. The patient’s vital signs and laboratory test results

Tests	Results	Reference ranges
Vital signs		
Axillary temperature, °C	39	36.0–37.0
Pulse, beats/min	126	60–100
Respiration rate, breaths/min	40	12–20
Systolic blood pressure, mmHg	62	90–139
Diastolic blood pressure, mmHg	35	60–89
Laboratory blood tests		
Pulse oxygen saturation, %	86.2	94–100
Full range C-reactive protein, mg/L	309.57	0.2–4.0
Leukocyte count, $\times 10^9$/L	11.56	3.5–9.5
Platelet count, $\times 10^9$/L	7	125–350
Fasting blood glucose, mmol/L	5.06	3.9–6.1
Activated partial thromboplastin time, s	108.2	21–34
Glomerular filtration rate, mL/min	22	>85
N-terminal pro-B-type natriuretic peptide, pg/mL	>10,000	0–125
Procalcitonin, ng/mL	>150	<0.10
Albumin, g/L	25	40–55
Globulin, g/L	20.7	20–40
Gram-negative lipopolysaccharides, pg/mL	210.2	0–10
Creatinine, µmol/L	201	57–97

Appendix Table 2. Antimicrobial susceptibility test results for *Burkholderia thailandensis* strain HNBT001 isolated from patient

Antimicrobial agent	MIC, μg/mL	Sensitivity
Ceftazidime	≤1	Susceptible
Imipenem	≤1	Susceptible
Trimethoprim/sulfamethoxazole	≤2/38	Susceptible
Tetracycline	≤4	Susceptible
Doxycycline	≤4	Susceptible

Appendix Table 3. Occurrence and functional characterization of virulence determinants for *Burkholderia thailandensis* strain HNBT001 isolated from patient*

Locus tags†	VFDB ID	VF	Description	VF category
AB9075_RS00230–60	VFG002490–6	Flagella, VF0430	Polar flagella required for motility and macrophage invasion	Motility
AB9075_RS01095–1100, AB9075_RS01150–1200, AB9075_RS01225–45	VFG002519–36	Flagella, VF0430	Polar flagella required for motility and macrophage invasion	Motility
AB9075_RS03035–45	VFG002426–8	Type IV pili, VF0431	Plays a role in adherence	Adherence
AB9075_RS03990, AB9075_RS04030–5	VFG002545–6, VFG002569	Capsule I, VF0436	Key virulence determinant; loss of capsule production results in severe attenuation in animal models of disease	Immune modulation
AB9075_RS06430	VFG002544	Quorum sensing, VF0433	NA	Biofilm
AB9075_RS12750	VFG045467	CdpA protein, VF0432	A major cyclic di-GMP–specific phosphodiesterase regulating intracellular levels of cyclic di-GMP	Regulation
AB9075_RS15335	VFG002425	Type IV pili, VF0431	Plays a role in adherence	Adherence
AB9075_RS17360–5, AB9075_RS17375–415, AB9075_RS17425–30, AB9075_RS17600–30	VFG002498–506, VFG002508–18	Flagella, VF0430	Polar flagella required for motility and macrophage invasion	Motility
AB9075_RS19950	VFG041098	*Salmonella* centrisome island, VF0974	Responsible for secreting 2 antibacterial effector proteins, Tlde1 and Tae4	Effector delivery system
AB9075_RS23425–70	VFG002429–35, VFG042964–6	Type IV pili, VF0431	Plays a role in adherence	Adherence
AB9075_RS23610–5	VFG002541–2	Quorum sensing, VF0433	NA	Biofilm
AB9075_RS23685–90, AB9075_RS23700–65, AB9075_RS23775–865	VFG002458–72, VFG002438–56	Bsa protein T3SS, VF0428; Bsa T3SS	Delivering effector proteins into host cells to manipulate host cell functions	Effector delivery system

Locus tags†	VFDB ID	VF	Description	VF category
		secreted effectors, VF0710		
AB9075_RS23880–95, AB9075_RS23905, AB9075_RS23915–60	VFG002474–5, VFG002477–81, VFG002483, VFG002485–8, VFG041119–20	T6SS-1, VF0429	Essential for virulence and plays a critical role in the intracellular lifestyle of *B. pseudomallei*	Effector delivery system
AB9075_RS25480, AB9075_RS25500, AB9075_RS26750, AB9075_RS26765, AB9075_RS29740	VFG002537–40, VFG002543	Quorum sensing, VF0433	NA	Biofilm

*Virulence determinants were identified by using the Virulence Factors Database (*1*) with screening thresholds set at ≥80% sequence identity and ≥60% coverage. ID, identification; NA, not applicable; T3SS, type III secretion system; T6SS-1, type VI secretion system 1; VF, virulence factor; VFDB, virulence factors database.

†Locus tags for strain HNBT001 (GenBank assembly no. GCA_048688115.1).

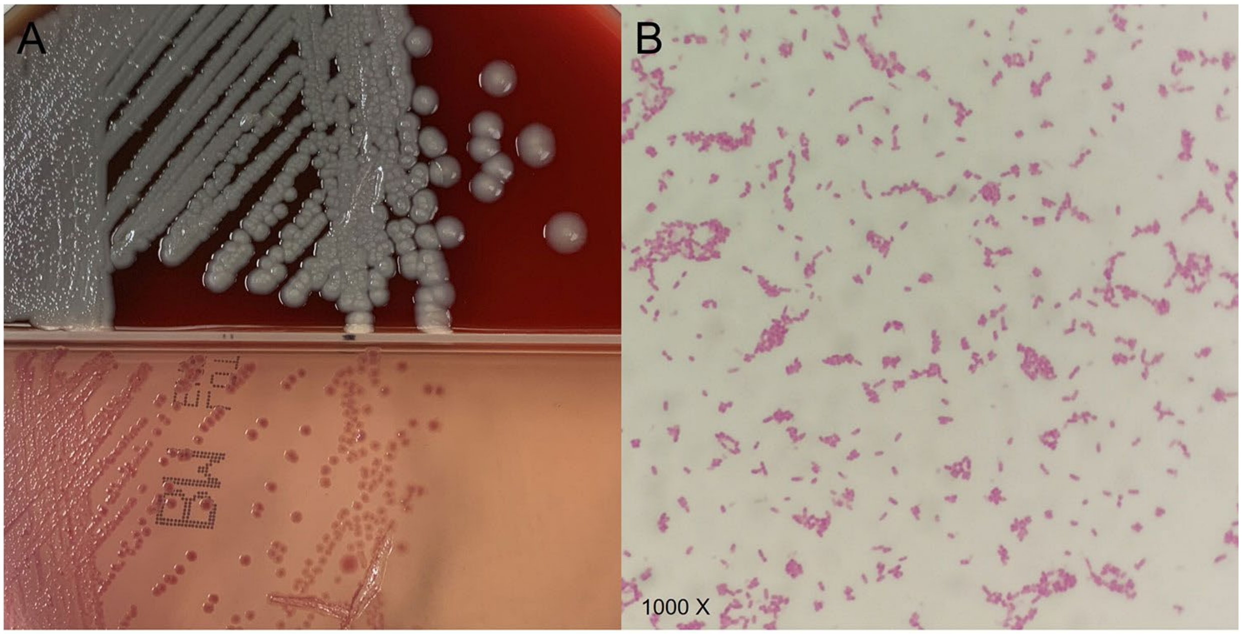

Appendix Figure 1. Colony morphology and gram-staining of *Burkholderia thailandensis* strain HNBT001 isolated from patient. A) Strain HNBT001 cultured for 48 hours on Columbia blood agar (upper) and MacConkey agar (lower) plates indicating colony morphology. B) Gram stain of smear from a colony of HNBT001 after 48 hour culture. Magnification is indicated.

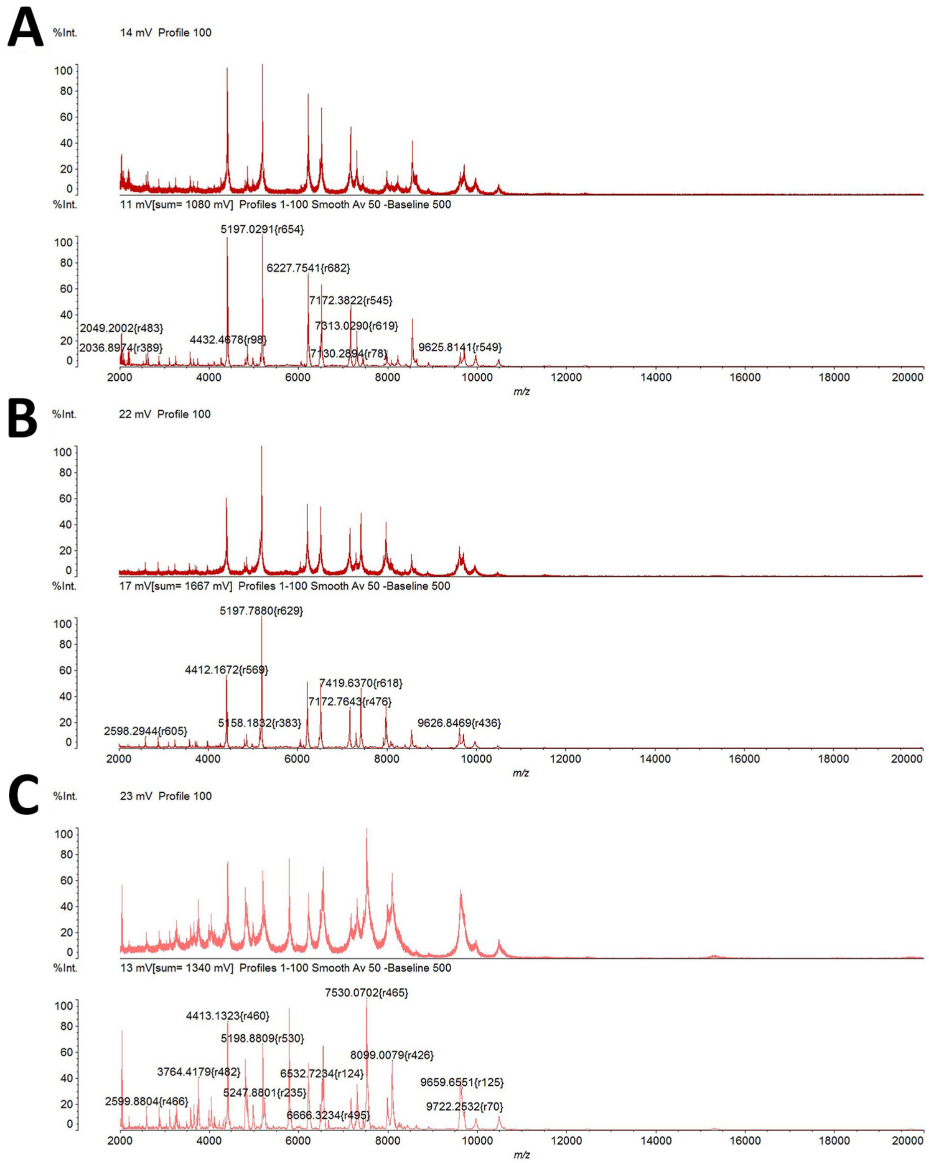

Appendix Figure 2. Matrix-assisted laser desorption/ionization time-of-flight mass spectrometry of *Burkholderia thailandensis* and *B. pseudomallei*. A) Mass spectrum of strain HNBT001 isolated from our patient; mass spectrometry could not identify the strain. Most proteins had a range of 2–12 kDa in molecular weight. Protein with the highest peak was 5.197 kDa, followed by 4.432 kDa and 7.172 kDa proteins. B) Mass spectrum of *B. thailandensis* (confirmed by whole-genome sequencing) from soil collected from Hele in Hainan Province, China; 3 proteins with the highest peaks were ≈5.197 kDa, 4.412 kDa, and 7.172 kDa. C) Mass spectrum of *B. pseudomallei* (confirmed by whole-genome sequencing) from a melioidosis patient; no peak was observed at 7.172 kDa.

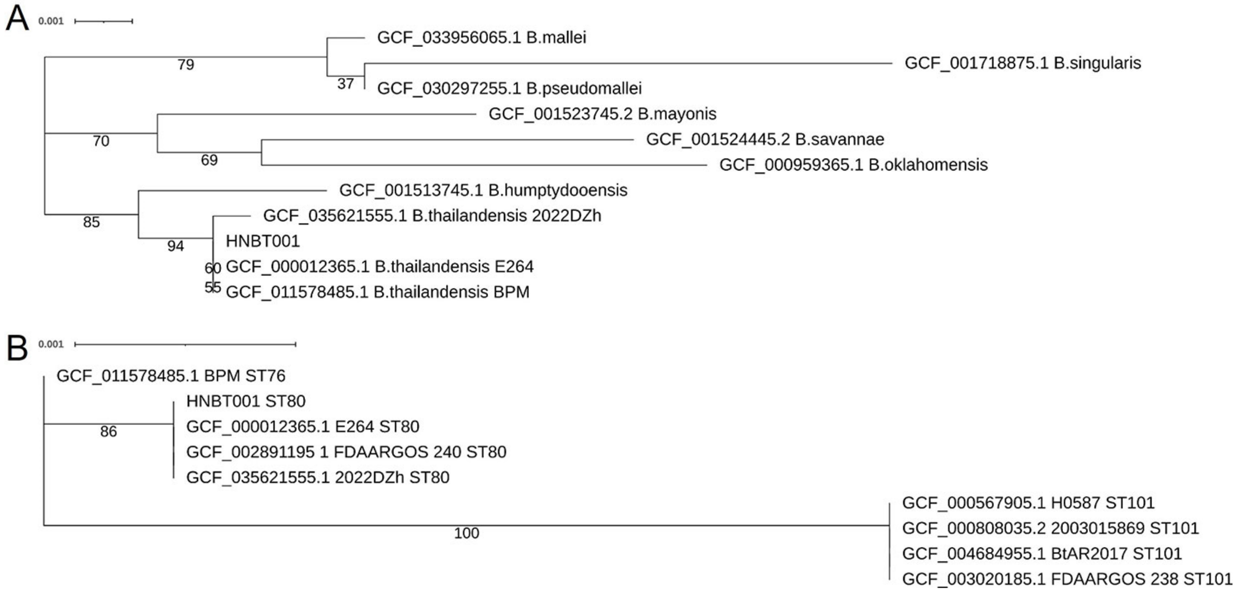

Appendix Figure 3. Phylogenetic analysis of *Burkholderia thailandensis* strain HNBT001 isolated from patient. Trees were constructed for the 16S rRNA sequence (A) and concatenated sequences of 7 household genes used for multilocus sequence typing (B). A) Tree indicated HNBT001 was homologous to other *B. thailandensis* strains according to 16S rRNA sequences. The reference genome, *B. thailandensis* E264, 2 previously isolated human-derived *B. thailandensis* strains from China, and reference genomes of 7 *B. pseudomallei* complex species were also included. B) Tree of concatenated sequences of 7 household genes. In addition to HNBT001, the reference genome, *B. thailandensis* E264, and 7 human-derived *B. thailandensis* strains from GenBank were also used for tree construction. Strain STs were classified according to pubMLST (*2*) and appended after strain names. Both trees were generated by using MEGA11 (*3*) with the following parameters: Kimura 2-parameter substitution model and 1,000 bootstrap replicates. Bootstrap values are indicated at the nodes. RefSeq https://www.ncbi.nlm.nih.gov/refseq) accession numbers are annotated in the corresponding strain labels. ST, sequence type.

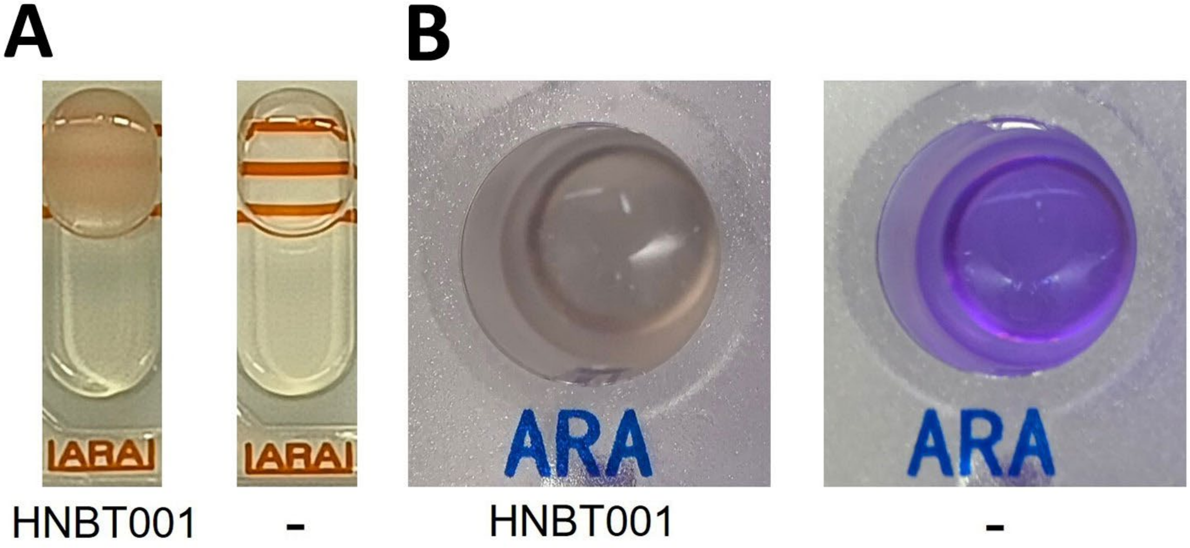

Appendix Figure 4. Arabinose assimilation test for *Burkholderia thailandensis* strain HNBT001 isolated from patient. A) Biochemical result of arabinose assimilation test by using API 20 NE strips (bioMérieux; https://www.biomerieux.com). HNBT001 was positive (turbid). B) Arabinose assimilation was positive (light yellow) for HNBT001 by using the DL-96E system (Zhuhai DL Biotech Co., Ltd., https://www.medicaldl.com). *B. thailandensis* can be distinguished from *B. pseudomallei* by its ability to assimilate arabinose. *B. pseudomallei* strain HNBP001 was used as the negative control for each test. ARA, arabinose.

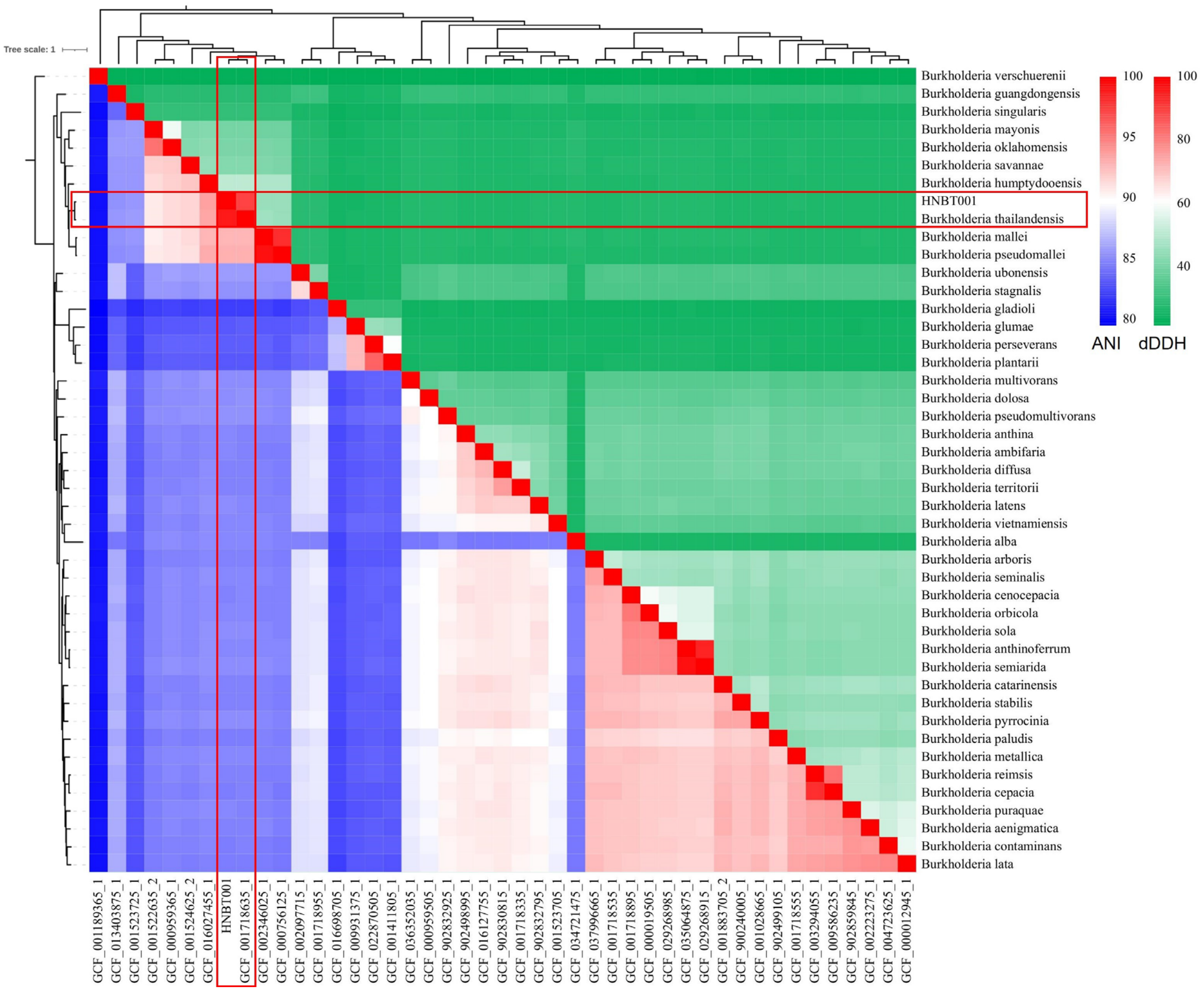

Appendix Figure 5. Phylogenetic analysis and pairwise comparisons according to both ANI and dDDH (formula d4) values of *Burkholderia thailandensis* strain HNBT001 and 44 representative genomes from *Burkholderia* spp. Tree was constructed by using FastTree (https://github.com/morgannprice/fasttree) and single-nucleotide polymorphisms matrix generated from genome sequences by using kSNP (*4*). Same tree clustering was used for both rows (labeled with *Burkholderia* spp. name) and columns (ignored branch lengths, labeled with GenBank accession nos.) in the heatmap. Colors indicate % ANI or % dDDH (red indicates 100% similarity between sequences). ANI, average nucleotide identity; dDDH, digital DNA–DNA hybridization.

References

1. Zhou S, Liu B, Zheng D, Chen L, Yang J. VFDB 2025: an integrated resource for exploring anti-virulence compounds. Nucleic Acids Res. 2025;53:D871–7. PubMed https://doi.org/10.1093/nar/gkae968

2. Jolley KA, Bray JE, Maiden MCJ. Open-access bacterial population genomics: BIGSdb software, the PubMLST.org website and their applications. Wellcome Open Res. 2018;3:124. PubMed https://doi.org/10.12688/wellcomeopenres.14826.1

3. Tamura K, Stecher G, Kumar S. MEGA11: molecular evolutionary genetics analysis version 11. Mol Biol Evol. 2021;38:3022–7. PubMed https://doi.org/10.1093/molbev/msab120

4. Gardner SN, Slezak T, Hall BG. kSNP3.0: SNP detection and phylogenetic analysis of genomes without genome alignment or reference genome. Bioinformatics. 2015;31:2877–8. PubMed https://doi.org/10.1093/bioinformatics/btv271
